# Clinical features of atypical tuberculosis mimicking bacterial pneumonia

**DOI:** 10.1515/med-2021-0349

**Published:** 2021-10-27

**Authors:** Min Qi, Pei-Jun Li, Ye Wang, Zong-An Liang

**Affiliations:** Department of Respiratory and Critical Care Medicine, West China Hospital, Sichuan University, Chengdu, 610041, China

**Keywords:** aPTB, bacterial pneumonia, radiological features

## Abstract

**Objectives:**

The aim of this study is to investigate clinical features of atypical pulmonary tuberculosis (aPTB) mimicking bacterial pneumonia, determine imaging features with the highest degree of correlation, and identify predictors for acid-fast bacilli (AFB) positivity.

**Methods:**

The clinical data of 259 patients considered as aPTB were retrospectively analyzed. The correlation of CT patterns was evaluated with Spearman analysis, and the predictors for AFB positivity were assessed with the multivariate analysis.

**Results:**

The most common symptom of aPTB was cough (84.6%), followed by fever and anorexia (47.1 and 41.7%, respectively). Infiltrated patchy was the most common radiological pattern (84.9%), followed by nodules (3–10 mm), micronodules (<3 mm), and consolidation (79.2, 78.8, and 66.0%, respectively). Nodules (3–10 mm) and micronodules (*r* = 0.988, *p* < 0.001), consolidation and air bronchogram (*r* = 0.590, *p* < 0.001), and pulmonary atelectasis and consolidation (*r* = 0.323, *p* < 0.001) showed high correlation. In the multivariate analysis, hyperpyrexia (OR, 2.29; 95% CI, 1.22–4.29) and bronchiectasis (OR, 2.06; 95% CI, 1.04–4.06) were the predictors of AFB-smear positivity, while bulla (OR, 0.22; 95% CI, 0.05–0.97) was the predictor of AFB-smear negativity.

**Conclusion:**

This study demonstrated the clinical and radiological features of aPTB mimicking pneumonia. Several paired radiological findings may guide us to the diagnosis of aPTB. Hyperpyrexia and bronchiectasis may be helpful for predicting AFB positivity, and bulla may be a predictive sign of AFB negativity.

## 1 Introduction

Pulmonary tuberculosis (PTB) is an infectious disease caused by *Mycobacterium tuberculosis*, remaining one of the top 10 leading threatening agents of death worldwide, especially in the underdeveloped countries and districts [1], which leads to overwhelming economic burden and persistent public health concern. The typical radiographic manifestations of PTB, such as upper lobe or superior-segment lower lobe fibro-cavitary pattern, give clues to the diagnosis of PTB [2]. However, some PTB patients presented with atypical symptoms and atypical chest images and have been mistaken for bacterial pneumonia when admission [3], leading to the delayed diagnosis and isolation of PTB patients [4].

PTB is a common cause of community-acquired pneumonia (CAP), and differential diagnosis between bacterial pneumonia and atypical PTB remains a great challenge for clinical physicians [3,5,6]. Yoon et al. reported that neutrophil–lymphocyte count ratio obtained at the initial diagnostic stage was a useful marker in discriminating PTB from bacterial CAP in an intermediate TB-burden country [7]. Similarly, Ugajin et al. demonstrated that serum procalcitonin (<0.5 ng/mL) in HIV-negative PTB patients was useful in the differential diagnosis of PTB and bacterial CAP [8]. However, these two studies did not describe the CT features in the study population. Kang et al. showed the clinical characteristics of 57 bacterial CAP patients and 30 PTB patients and found that compared with bacterial CAP patients, PTB patients presented a higher proportion of cavitary lesions and upper lobe dominance in CT images [9]. Matsuura and Yamaji reported a case with consolidation and multicavity lesions in chest radiography, which was diagnosed as bacterial pneumonia initially but finally confirmed as PTB by AFB smear and polymerase chain reaction test after the failure of antibiotic therapy [10]. Jacobs et al. reported that 32 children with the primary diagnosis of bacterial necrosis pneumonia were found to have tuberculosis per tuberculosis culture and AFB smear [11]. Nonetheless, there are limited studies exploring the clinical and radiological features of atypical PTB (aPTB) mimicking bacterial pneumonia in adults.

To improve the early diagnostic accuracy of aPTB mimicking bacterial pneumonia, the current retrospective study was conducted to analyze the most frequent clinical and radiological findings in 259 patients with initially presumptive diagnosis of bacterial pneumonia but without response to first-line antibiotics and finally confirmed with PTB by percutaneous transthoracic needle biopsy or transbronchial lung biopsy. In addition, we aimed to determine imaging features with the highest degree of correlation and identify the predictors for acid-fast bacilli (AFB) positivity.

## 2 Methods

### 2.1 Patients

We retrospectively reviewed the electronic medical database of patients admitted to our hospital from January 2012 to December 2019.

**Figure 1:**
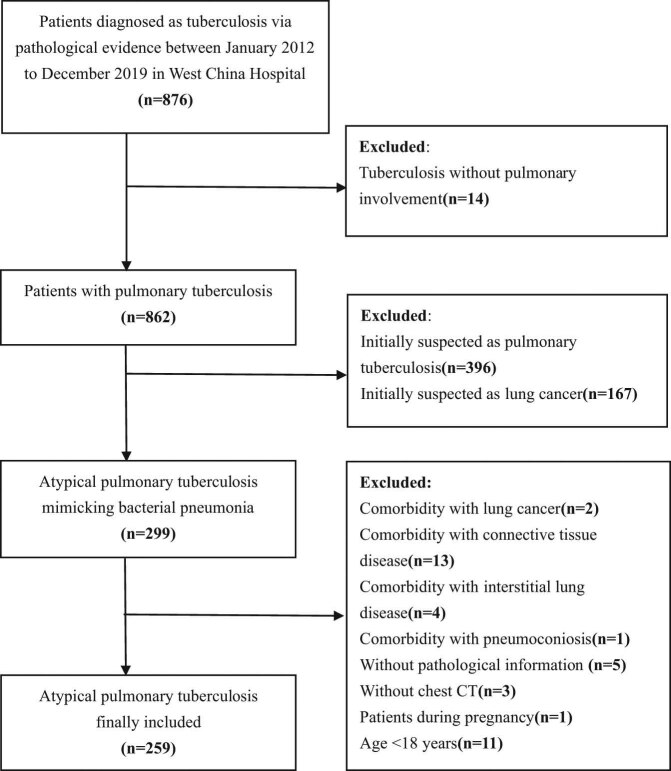
The flowchart of patients’ enrollment. There were 876 patients diagnosed as tuberculosis pathologically, and after excluding those patients who did not meet the criteria, 259 patients were enrolled eventually.


The patients who met all the following criteria were screened in this study: (1) a presumptive diagnosis of bacterial pneumonia, (2) no response to first-line antibiotics, and (3) a confirmed diagnosis of tuberculosis by the percutaneous transthoracic needle biopsy or transbronchial lung biopsy with the pulmonary tissue tested positive for *M. tuberculosis* DNA by gene amplification. Failure to antimicrobial treatment was defined as persistent fever (>38°C) and/or clinical symptoms (malaise, cough, expectoration, and dyspnea) after at least 72 h of antimicrobial treatment [12]. The exclusion criteria were as follows: (1) extrapulmonary tuberculosis without pulmonary involvement, (2) the initial diagnosis was PTB or lung cancer, (3) PTB with comorbidities of other chronic lung diseases (like lung cancer, connective tissue disease, interstitial lung disease, and pneumoconiosis), (4) incomplete medical records, (5) without chest CT performed in our hospital, (6) patients during pregnancy, or (7) age <18 years. Finally, 259 patients with aPTB mimicking bacterial pneumonia were included for analysis eventually (Figure 1).

**Ethics approval and consent to participate:** This retrospective study protocol was approved by the Institutional Ethics Committee at West China Hospital, Sichuan University (No. 2020-404). Due to the retrospective nature of the study, informed consent was waived.

### 2.2 Data collection

The following data in medical records were collected: (1) demographic data, such as age, gender, body mass index, smoking status, underlying diseases, drugs, and alcohol abusing; (2) the onset signs and symptoms including cough, purulent sputum, hemoptysis, fever (<39℃), hyperpyrexia (≥39℃), night sweating, asthenia, weight loss, chest pain, dyspnea, and anorexia; and (3) microbiological data (AFB stain, culture results, gene amplification test performed on sputum or bronchoalveolar lavage).

### 2.3 CT technique and imaging analysis

CT scanning was performed in all the 259 patients, ranging from 16-detector to 128-detector CT scanners (Philips Medical Systems, Best, the Netherlands or Siemens Medical Systems, Erlangen, Germany). Scanning was performed from the level of the superior margin of the thoracic cavity to the level of the inferior margin of the thoracic cavity. In all the patients, CT scans were obtained with 5-mm-thick sections at 5 mm intervals. Scanning parameters were 100–120 kVp and 70–200 mA.

The presence or absence of the following lung parenchymal manifestations and lymph node findings was recorded. CT imaging was analyzed for major pulmonary lesions according to the definition by the Fleischner Society [13]. The nodule was defined as a rounded or irregular opacity, well or poorly defined, measuring up to 30 mm in diameter. According to different sizes, nodules were divided into micronodules (diameter <3 mm), nodules >10 mm, and nodules between 3 and 10 mm. Bronchiectasis referred to irreversible localized or diffuse bronchial dilatation. Pulmonary atelectasis was defined as reduced pulmonary volume, accompanied by increased opacity or attenuation in the affected part of the lung. Consolidation was defined as the homogeneous increase in pulmonary parenchymal attenuation that obscured the margins of vessels and airway walls. Air bronchogram was a pattern of air-filled bronchi on a background of the opaque airless lung. Tree-in-bud (TIB) referred to multiple areas of centri-lobular nodules with a linear branching pattern that resembled a budding tree. The cavity was defined a gas-filled space, seen as a lucency or low-attenuation area, within pulmonary consolidation, a mass, or a nodule. Ground glass opacities were defined as an area of hazy increased lung opacity with preservation of bronchial and vascular margins. Calcification referred to a zone of lung parenchyma with a very high attenuation value. Bulla referred to airspace measuring more than 10 mm in diameter sharply demarcated by a thin wall no greater than 1 mm in thickness. Other radiological patterns like narrowed lumen, fibrous pattern, pleural thickening or pleural effusion, reticular opacities, and infiltrated patchy and the mediastinal lymph node findings including calcifications, cavity, colliquation, and lymphadenopathy were recorded.

### 2.4 Statistical analyses

Statistical analyses were conducted with the IBM SPSS statistics ver. 23 software package (IBM SPSS, NY, USA). The correlation between CT findings was performed by Spearman correlation analysis. The association between AFB and clinical characteristics and each image finding were assessed by the univariate analysis. The variables with a *p* value <0.1 in the analysis were then entered into a multivariate logistic regression analysis to assess the additive effects of variables for AFB. The results were expressed as odds ratio (OR) of being AFB positive (with 95% confidence interval [CI]). *p* < 0.05 (two-tailed) indicated that there was statistical significance.

## 3 Results

We enrolled 259 patients in this retrospective study (Table 1), with 150 men (57.9%) and 109 women (42.1%), ranging from 18 to 84 years (mean, 48.5 years). The average body mass index was 19.9 kg/m^2^, and the most frequent comorbidity was diabetes mellitus (14.7%), followed by chronic obstructive pulmonary disease (9.3%), glucocorticoid using (6.7%), malignant tumors (5.8%), transplantation-related immunosuppression (4.25%), chronic renal failure needing dialysis (2.7%), HIV infection (0.1%), illicit drug (0.1%), alcohol using (0.04%), and treatment with TNF-α inhibitors (0.04%).

**Table 1:** General characteristics of patients with atypical pulmonary tuberculosis mimicking pneumonia

Characteristics	*n* (%)
Age, mean ± SD, year	48.5 ± 18.9
Male, sex	150(57.9)
Body mass index, mean ± SD, kg/m^2^	19.9 ± 3.0
Comorbidity	
Diabetes mellitus	38(14.7)
Malignant tumors	15(5.8)
HIV	2(0.1)
Transplantation-related immunosuppression	11(4.2)
Chronic renal failure needing dialysis	7(2.7)
Chronic obstructive pulmonary disease	24(9.3)
Illicit drug using	2(0.1)
Alcohol abusing	1(0.04)
Treatment with glucocorticoids	17(6.7)
TNF-α inhibitors	1(0.04)

**Figure 2:**
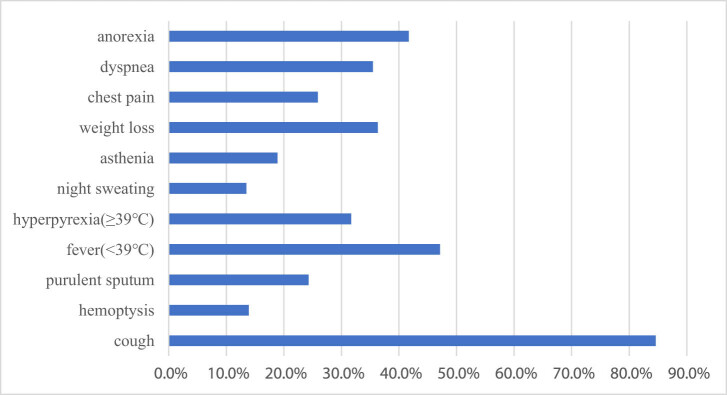
The frequency of symptoms. Symptoms varied widely in patients with atypical pulmonary tuberculosis mimicking bacteria pneumonia. The most common symptom reported was cough (84.6%), while the least common symptom was night sweating (13.5%).

The most common symptoms reported were cough (84.6%), fever (<39℃) (47.1%), anorexia (41.7%), weight loss (36.3%), and dyspnea (35.5%), whereas the less common symptoms were hyperpyrexia (≥39℃; 31.7%), chest pain (25.9%), purulent sputum (24.3%), asthenia (18.9%), hemoptysis (13.9%), and night sweating (13.5%; Figure 2).

### 3.1 Frequency of radiological features

**Figure 3:**
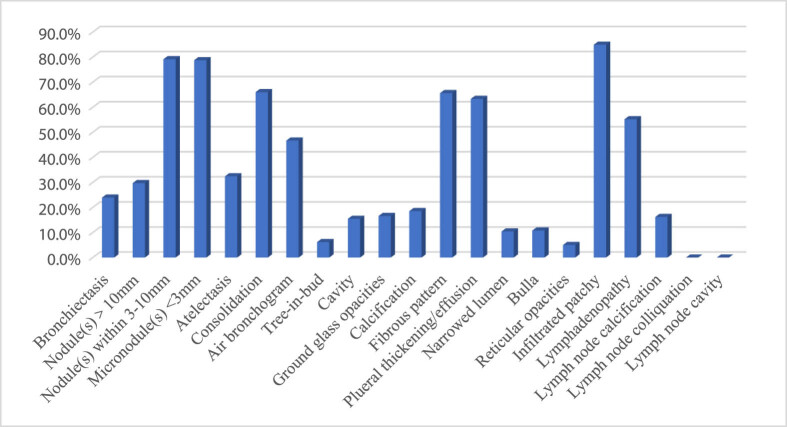
The frequency of radiological features. The radiological findings in patients with atypical pulmonary tuberculosis mimicking bacteria pneumonia were diverse. The relatively common manifestations were infiltrated patchy (84.9%), nodules within 3–10 mm (79.2%), micronodules (78.8%), consolidation (66.0%), fibrous pattern (65.6%), and pleural thickening/effusion (63.3%).

The frequency of the chest CT image findings is shown in Figure 3. The relatively common manifestations were infiltrated patchy (84.9%), nodules within 3–10 mm (79.2%), micronodules (78.8%), consolidation (66.0%), fibrous pattern (65.6%), and pleural thickening/effusion (63.3%). Lymph node colliquation and cavity were not shown in our included patients.

### 3.2 Correlation of radiological findings

There were 38 couples of radiological findings demonstrated correlation (*p* < 0.05) in total. Only three pairs showed relatively high correlation (*r* ≥ 0.03). Nodules within 3–10 mm and micronodules showed the highest correlation (*r* = 0.988, *p* < 0.001), followed by consolidation and air bronchogram (*r* = 0.590, *p* < 0.001) and pulmonary atelectasis and consolidation (*r* = 0.323, *p* < 0.001). No TIB findings showed correlation with the other radiological findings.

### 3.3 Symptoms and radiological findings in AFB staining

In the univariate analysis (Table 2), we found that only hyperpyrexia (≥39℃) had a positive predictive value for AFB staining. Those clinical manifestations and radiological findings (fever, anorexia, bronchiectasis, air bronchogram, pleural thickening/effusion, and bulla) with *p* < 0.1 were enrolled into the multivariate analysis. Finally, hyperpyrexia (≥39℃) and bronchiectasis showed significant relations with AFB positivity (OR, 2.29; 95% CI, 1.22–4.29, and OR, 2.06; 95% CI, 1.04–4.06). However, the bulla indicated AFB negativity, with OR value of 0.22 (95% CI, 0.05–0.97).

**Table 2:** The symptoms and radiological findings in different AFB status

	AFB positive	AFB negative	Univariate	Multivariable	
Symptoms and images	(*N* = 55) No. (%)	(*N* = 204) No. (%)	*p* value	*p* value	OR (95%CI)
Cough	45 (81.8)	174 (85.3)	0.527		
Hemoptysis	11 (20)	25 (12.3)	0.141		
Purulent sputum	16 (29.1)	47 (23.0)	0.353		
Fever (<39℃)	32 (58.2)	90 (44.1)	0.064		
Hyperpyrexia (≥39℃)	25 (45.5)	57 (27.9)	0.013	0.010	2.29 (1.22–4.29)
Night sweating	8 (14.5)	27 (13.2)	0.801		
Asthenia	12 (21.8)	37 (18.1)	0.536		
Weight loss	18 (32.7)	79 (38.7)	0.415		
Chest pain	14 (25.5)	53 (26.0)	0.937		
Dyspnea	18 (32.7)	74 (36.3)	0.626		
Anorexia	29 (52.7)	79 (38.7)	0.062		
Bronchiectasis	18 (32.7)	44 (21.6)	0.085	0.038	2.06 (1.04–4.06)
Nodule(s) >10 mm	15 (27.3)	64 (31.4)	0.434		
Nodule(s) (3–10 mm)	46 (83.6)	161 (78.9)	0.583		
Micronodule(s)	45 (81.8)	159 (77.9)	0.533		
Atelectasis	18 (32.7)	66 (32.4)	0.958		
Consolidation	39 (70.9)	132 (64.7)	0.389		
Air bronchogram	32 (58.2)	89 (43.6)	0.055		
Tree in bud	2 (3.6)	14 (6.9)	0.571		
Cavity	11 (20)	30 (14.7)	0.340		
Ground glass opacities	12 (21.8)	31 (15.2)	0.241		
Calcification	13 (23.6)	35 (17.2)	0.272		
Fibrous pattern	32 (58.2)	138 (67.6)	0.190		
Pleural thickening/effusion	29 (52.7)	134 (65.7)	0.077		
Narrowed lumen	3 (5.5)	24 (11.8)	0.174		
Bulla	2 (3.6)	26 (12.7)	0.054	0.046	0.22 (0.05–0.97)
Reticular opacities	2 (3.6)	14 (6.9)	0.571		
Infiltrated patchy	50 (90.9)	170 (83.3)	0.163		
Lymphadenopathy	30 (54.5)	115 (56.4)	0.676		
Lymph node calcification	10 (18.2)	32 (15.9)	0.656		
Lymph node colliquation	0 (0)	0 (0)	NA		
Lymph node cavity	0 (0)	0 (0)	NA		

AFB = acid-fast bacilli, CI = confidence interval, NA = not available, OR = odds ratio.

## 4 Discussion

In this study, we were able to demonstrate the clinical and radiological features of aPTB mimicking bacterial pneumonia. Several paired radiological findings may guide us to the diagnosis of aPTB mimicking bacterial pneumonia. Nodules within 3–10 mm and micronodules showed the highest correlation in those patients, followed by consolidation and air bronchogram, pulmonary atelectasis, and consolidation. Hyperpyrexia (≥39℃) and bronchiectasis were the predictors of AFB positivity, and bulla was the predictor of AFB negativity.

Risk factors like diabetes mellitus, chronic obstructive pulmonary disease, usage of glucocorticoid, malignant tumors, transplantation-related immunosuppression, chronic renal failure needing dialysis, HIV infection, illicit drug and alcohol usage, and treatment with TNF-α inhibitors were reported as comorbidities in aPTB [14]. Previous studies have confirmed that those related diseases were the predisposing factors of aPTB and could cause cell-mediated immunity defects, leading to the atypical radiological manifestations [14,15,16,17].

The most common presenting symptoms of aPTB patients in this study were cough, fever (<39℃), anorexia, weight loss, and dyspnea (84.6, 47.1, 41.7, 36.3, and 35.5%, respectively), which corresponded to aPTB symptoms [18,19]. However, hemoptysis was also one of the frequent symptoms in typical PTB [20], which only presented in 13.9% patients in our study. Lau et al. analyzed 97 smear-positive PTB cases and found that typical PTB was more likely to have cavitation lesions [21]. Cavitation is associated with hemoptysis, and the presence of cavitation may give clues to the suspected diagnosis of PTB and early treatment [22]. Therefore, aPTB with less hemoptysis incidence would delay the diagnosis of PTB to some extent.

We found that the radiological features of aPTB were diverse, and the relatively common manifestations were infiltrated patchy, nodule(s) within 3–10 mm, micronodule(s), consolidation, fibrous pattern, pleural thickening/effusion, lymphadenopathy, and air bronchogram (84.9, 79.2, 78.8, 66.0, 65.6, 63.3, 55.2, and 46.7%, respectively). Generally, infiltrated patchy, nodules, cavitation, and unilateral pleural effusion were considered as typical radiological manifestations of PTB, while the airspace consolidation was evaluated as atypical manifestations of PTB [23]. A retrospective study of 66 non-AIDS patients showed that patients with risk factors were more likely to progress rapidly to active diseases with atypical radiology, such as parenchymal consolidation, and increased nodules [24]. Cavitation is a common finding in typical PTB, seen in 20–45% of patients on chest images [19]. However, in immunocompromised patients, the cavitation lesion is less seen due to the weakened functions of lymphocytes and macrophage cells recruited to form granulomatous lesions [16].

Besides, some correlations between radiological findings were discovered, among them, nodule(s) within 3–10 mm and micronodule(s) with the highest correlation (*r* = 0.988, *p* < 0.001), followed by consolidation and air bronchogram (*r* = 0.590, *p* < 0.001), pulmonary atelectasis, and consolidation (*r* = 0.323, *p* < 0.001). Tuberculoma can be seen in about 5% patients in postprimary tuberculosis, and even as the main or only abnormality on chest radiographs [25]. The manifestations were solitary or multiple modules or masses ranging from 0.5 to 40 mm or greater in diameter, and satellite lesions were seen in up to 80% of cases [26]. The high correlation between nodule(s) within 3–10 mm and micronodule(s), to some extent, could be considered as tuberculoma and its satellite lesions. Besides, multiple, ill-defined nodules within 10 mm distributed in segmental or lobar distribution is the manifestation of bronchogenic spread of PTB [27], and the typical manifestation in high-resolution CT is the TIB pattern [28]. Dense and homogeneous parenchymal consolidation with air bronchogram is a common manifestation of parenchymal lesions of primary tuberculosis, generally without lobar predilection and more than one pulmonary segment involved [19], and the predominance in the middle and lower lobes is suggestive of PTB [28]. Atelectasis is also a frequent finding in postprimary tuberculosis, which marks fibrotic response, with retraction of the hilum, compensatory hyperinflation in corresponding lobes, and mediastinal shift toward the fibrotic lung [26]. Residual parenchymal scarring, a form of atelectasis, can be seen at sites of prior consolidation after resolution [19]. The coexistence of pulmonary atelectasis and consolidation may suggest the reinfection of PTB with compromised immunity. Collectively, those paired manifestations detected were seen as patterns with positive predictive value for PTB.

So far, the AFB smear remains the mainstay diagnostic method for PTB in many TB endemic countries. Even though with higher specificity, the sensitivity is about 20–60% [29,30,31] and that was in line with the positivity of AFB 21.1% (55/259) in our study. The low sensitivity of AFB-smear makes the diagnosis of PTB challenging, especially in PTB with atypical manifestations. In our study, we attempted to analyze the differences between AFB smear positive and negative to find some clinical and radiological features of predictive value for AFB smear. After multiple variable regression analysis, hyperpyrexia (≥39℃) and bronchiectasis showed positive predicted value for AFB smear, and on the contrary, the bulla demonstrated a negative predicted value. Fever, especially low-grade intermittent fever, gives a clue to tuberculosis, and high-grade fever with a long duration may indicate a great load of *Mycobacterium tuberculosis* and vigorous immune response. Bronchiectasis can occur after a prior episode of PTB, and the pathophysiological changes of bronchiectasis, like increased mucus secretion, decreased mucociliary clearance, airway wall thickening, and transient collapse of weakened dilated airways, make the pathogens colonization more easily [32,33]. When *Mycobacterium tuberculosis* becomes active under the immunocompromised situation, persistent cough and sputum make the higher PTB smear possible. Cystic lesions, similar to bulla in radiology, were more frequently with sputum-negative PTB in a prospective observational study enrolled 147 consecutive PTB patients [30], and the exact mechanism needs further investigation.

Our study has several limitations. First, it was a single-center retrospective descriptive study, lacking of control cases that limited the findings to generalization. Second, we did not describe the detailed pulmonary lobes involved, which would make the accurate localization of pulmonary lesions when confronted with similar radiological patterns. Finally, patients with bacterial infections at the same time were not ruled out, which would have an influence on the analysis theoretically. However, the enrolled patients underwent standard antibacterial courses, which would decrease the effects caused by bacteria to some extent. In the future, a case–control study with large samples is needed to find out the difference between aPTB mimicking bacterial pneumonia and bacterial pneumonia and to establish a risk-factor model for aPTB.

## 5 Conclusion

Our study demonstrated the clinical and radiological features of aPTB mimicking bacterial pneumonia. Nodules and micronodules, consolidation and air bronchogram, and pulmonary atelectasis and consolidation showed high correlations, which may help clinicians to early identify aPTB mimicking bacterial infection. Hyperpyrexia (≥39℃) and bronchiectasis may be positive predictive value for AFB smear, while bulla may be negative predictive value for AFB smear.

## Abbreviations

aPTB: atypical pulmonary tuberculosis
AFB: acid-fast bacilli
CAP: community-acquired pneumonia
CI: confidence interval
OR: odds ratio
PTB: pulmonary tuberculosis
TIB: tree-in-bud
